# Pomegranate Protection against Cardiovascular Diseases

**DOI:** 10.1155/2012/382763

**Published:** 2012-11-18

**Authors:** Michael Aviram, Mira Rosenblat

**Affiliations:** The Lipid Research Laboratory, Technion Faculty of Medicine, The Rappaport Family Institute for Research in the Medical Sciences and Rambam Medical Center, Haifa 31096, Israel

## Abstract

The current paper summarizes the antioxidative and antiatherogenic effects of pomegranate polyphenols on serum lipoproteins and on arterial macrophages (two major components of the atherosclerotic lesion), using both *in vitro* and *in vivo* humans and mice models. Pomegranate juice and its by-products substantially reduced macrophage cholesterol and oxidized lipids accumulation, and foam cell formation (the hallmark of early atherogenesis), leading to attenuation of atherosclerosis development, and its consequent cardiovascular events.

## 1. Polyphenolic Flavonoids and Cardiovascular Diseases

Polyphenolic flavonoids compose the largest and the most studied group of plant phenolics. Over 4000 different flavonoids have been identified to date. Flavonoids are grouped into anthocyanins and anthoxanthins. Anthocyanins are glycosides of anthocyanidin, and they are the most important group of water-soluble plant pigments, responsible for the red, blue, and purple colors of flowers and fruits. Anthoxanthins are colorless or colored white-to-yellow, and include flavonols, flavanols, flavones, flavans, and isoflavones. Flavonoids are powerful antioxidants, and their activity is related to their chemical structure [1, 2]. Plant flavonoids can act as potent inhibitors of low-density lipoprotein (LDL) oxidation [3, 4] or of macrophage oxidation [5]. Dietary consumption of flavonoids was shown to be inversely related to morbidity and mortality from coronary heart disease (CHD) [6]. Moreover, an inverse association between flavonoid intake and subsequent occurrence of ischemic heart disease, or cerebrovascular disease was shown [7, 8]. Reduced morbidity and mortality from cardiovascular diseases, in spite of high intake of saturated fat among French, the so-called French paradox [9], has been attributed to the regular intake of red wine in the diet. Dietary consumption of flavonoid-rich nutrients, as well as pure flavonoids, was shown to attenuate the progression of atherosclerosis in animals [10]. Reduced development of atherosclerotic lesion areas in the atherosclerotic apolipoprotein E-deficient (E^0^) mice was demonstrated following consumption of red wine [11, 12] licorice root extract [13, 14], grape powder [15], or ginger extract [16].

## 2. Pomegranate Juice (PJ) Polyphenols Inhibit the Development of Atherosclerotic Lesion

The pomegranate tree, which is said to have flourished in the Garden of Eden, has been extensively used as a folk medicine in many cultures [17, 18]. Edible parts of pomegranate fruits (about 50% of total fruit weight) comprise 80% juice and 20% seeds. Fresh juice contains 85% moisture, 10% total sugars, 1.5% pectin, ascorbic acid, and polyphenols.

Content of soluble polyphenols in PJ varies within the limits of 0.2%–1.0%, depending on the variety, and includes mainly anthocyanins (such as cyanidin-3-glycoside, cyanidin-3, 3-diglycoside, and delphindin-3-glucoside) and anthoxanthins (such as catechins, ellagic tannins, and gallic and ellagic acids) [19, 20]. Ellagic acid and hydrolysable ellagitannins are both implicated in protection against atherogenesis, along with their potent antioxidant capacity. Punicalagin is the major ellagitannin in PJ, and this compound is responsible for the high antioxidant activity of this juice. As a major source for polyphenolics, PJ was shown to be a very potent antioxidant against LDL oxidation and, in parallel, to inhibit atherosclerosis development in mice and in humans [21–23]. *In vivo* studies were conducted first in order to evaluate whether the active antioxidant components of PJ are absorbed. Recent studies examined the bioavailability and metabolism of punicalagin in the rat as an animal model [24, 25]. Two groups of rats were studied. One group was fed with standard rat diet (*n* = 5), and the second one with the same diet plus 6% punicalagin (*n* = 5). The daily intake of punicalagin ranged from 0.6–1.2 g. Glucuronides of methyl ether derivatives of ellagic acid and punicalagin were detected in plasma. 6H-Dibenzo [b, d] pyran-6-one derivatives were also observed in the plasma, especially during the last few weeks of the study. In urine, the main metabolites observed were the 6H-dibenzo [b, d] pyran-6-one derivatives, and were present as aglycones or as glucuronides. It was concluded that since only 3%–6% of the ingested punicalagin was detected as such or as metabolites in urine and feces, the majority of this ellagitannin has to be converted to undetectable metabolites or accumulated in nonanalysed tissues. Only traces of punicalagin metabolites were detected in liver or kidney. In humans, following consumption of PJ (180 mL) containing 25 mg of ellagic acid and 318 mg of hydrolysable ellagitannins (as punicalagin), ellagic acid was detected in human plasma 1 hour after ingestion at a maximum concentration of 32 ng/mL, and by 4 hours it was completely eliminated [26]. Thus, active components of PJ are indeed absorbed, and subsequently affect biological processes which are related to atherogenesis protection. Upon analyzing the influence of the physiological conditions in the stomach and small intestine on pomegranate bioactive compounds bioavailability using an *in vitro* availability method, it was demonstrated that pomegranate phenolic compounds are available during digestion in a high amount (29%). Nevertheless, due to pH, anthocyanins are largely transformed into nonred forms or degraded [27].

### 2.1. Studies in Atherosclerotic Mice

**Figure 1:**
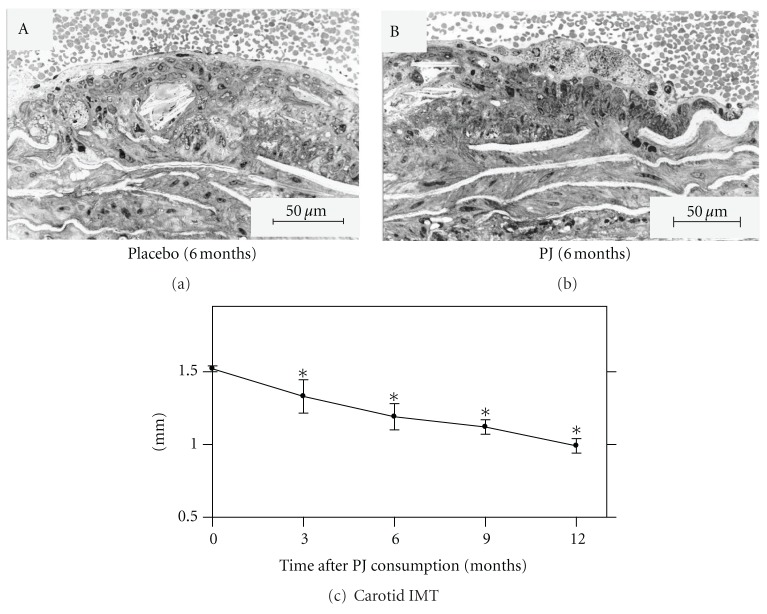
Pomegranate juice (PJ) consumption attenuates atherosclerotic lesion development in the atherosclerotic apolipoprotein E-deficient (E^0^) mice (a)-(b), or in patients with carotid artery stenosis (CAS, c). Twenty E^0^ mice, 4-month-old, with advanced atherosclerosis, or 10 patients with severe CAS were supplemented with PJ concentrate (12.5 *μ*L/mouse/day or 50 mL/day, resp.) for 9 weeks or for 1 year, respectively. Photomicrographs of typical foam cells from 6 months old E^0^ mice administered a placebo (a) or PJ (b) are presented. Effect of PJ consumption on human common carotid artery intima media thickness (IMT, c) is shown. *P < 0.01 (mean ± SEM, after PJ versus before PJ consumption).

PJ supplementation to the atherosclerotic E^0^ mice reduced the size of their atherosclerotic lesion and the number of foam cells in their lesion [28], in comparison to control placebo-treated E^0^ mice that were supplemented with water. We also analyzed the therapeutic potency of PJ by its administration to E^0^ mice with already advanced atherosclerosis. Atherosclerotic E^0^ mice at 4 months of age, were supplemented for 2 months with 31 *μ*L of PJ (equivalent to 0.875 *μ*moles of total polyphenols/mouse/day, which is equivalent to about one glass-8Oz/human/day), and were compared to age-matched placebo-treated mice. PJ supplementation to 4-month-old E^0^ mice was still able to inhibit the progression of the disease, as it reduced the mice atherosclerotic lesion size by 17%, in comparison to atherosclerotic lesion of the age-matched placebo-treated mice (Figures 1(a) and 1(b)) [29]. These results were further confirmed by de Nigris et al [30], who demonstrated that oral administration of PJ to hypercholesterolemic LDL-receptor deficient mice at various stages of the disease reduced significantly the progression of atherosclerosis. Thus, PJ exhibits preventive, as well as therapeutic effects against atherosclerosis.

### 2.2. Studies in Humans

Measurements of the arterial stiffness of the common carotid arteries in 73 patients with at least one cardiovascular risk factor that consumed PJ (Wonderful variety, 240 mL/day for one year) showed trends to increased elasticity in the PJ-treated group versus the placebo-treated group (who received beverage of similar caloric content, flavor and color, unpublished data). The effect of a daily consumption of PJ for 3 months on myocardial perfusion in 45 patients who had CHD was also studied. Patients were randomly assigned into one of two groups: a PJ group (240 mL/day) or a placebo group. The experimental and control groups showed similar levels of stress-induced ischemia at baseline. After 3 months however, the extent of stress-induced ischemia decreased in the pomegranate group, but increased in the control group. This benefit was observed without changes in cardiac medications, blood sugar, hemoglobin A1c, body weight, or blood pressure, in either group [31]. We next investigated the effects of PJ consumption by patients with carotid artery stenosis (CAS) on carotid lesion size, in association with changes in oxidative stress [32]. Ten patients were supplemented with PJ for up to one year, and nine CAS patients that did not consume PJ served as a control group. Blood samples were collected before treatment and after 3, 6, 9, and 12 months of PJ consumption. Patients' carotid intima-media thickness (CIMT) was compared between the PJ group and the control group. While in the control group (no PJ) CIMT increased by 10% after 1 year, PJ consumption resulted in a significant CIMT reduction, by up to 35%. Analysis of the mean CIMT (of the left and right common carotid arteries) before and during PJ consumption revealed a gradual reduction of 13%, 22%, 26%, and 35%, as observed after 3, 6, 9, and 12 months of PJ consumption, respectively, in comparison to baseline values (“0 time”, Figure 1(c)). On examination of the internal carotid arteries, flow velocities were calculated at the stenotic sites, and expressed by peak systolic velocity (PSV) and end diastolic velocity (EDV). The ultrasound outcome data were the change over time in maximal IMT, which was measured in the same preselected carotid artery segments. Twelve months of PJ consumption, resulted in PSV reduction by 12% and 28% in the left and the right carotid arteries, respectively. Mean carotid EDV of both left and right carotid arteries gradually decreased, by 16%, 20%, 31%, and 44%, after 3, 6, 9, and 12 months of PJ consumption, respectively. 

A randomized, double-blind trial assessed the influence of PJ consumption on anterior and posterior CIMT progression rates in subjects at moderate risk for coronary heart disease. Subjects were men (45 to 74 years old) and women (55 to 74 years old) with one or more major CHD risk factors and baseline posterior wall CIMT of 0.7 to 2.0 mm, without any significant stenosis. Participants consumed 240 mL/day of PJ (*n* = 146), or a control beverage (*n* = 143) for up to 18 months. No significant difference in overall CIMT progression rate was observed between PJ and control treatments. In exploratory analyses however, in subjects in the most adverse tertiles for baseline serum lipid peroxides, triglycerides (TGs), high-density lipoprotein (HDL) cholesterol, TGs/HDL cholesterol, total cholesterol/HDL cholesterol, and apolipoprotein-B100, those in the PJ group had significantly less anterior wall and/or composite CIMT progression versus control subjects. These results suggest that in subjects at moderate CHD risk, PJ consumption had no significant effect on overall CIMT progression rate but slowed CIMT progression in subjects with increased oxidative stress and disturbances in the TG-rich lipoprotein/HDL axis [33].

## 3. Antioxidative Properties of PJ

### 3.1. Antioxidative Capacity of PJ in Comparison to other Juices

PJ was shown to possess an antioxidant activity that was three times higher than the antioxidant activity of green tea [19]. The antioxidant activity was higher in juice extracted from whole pomegranate than that of juice obtained from arils only, suggesting that the processing extracts some of the hydrolyzable tannins present in the fruit rind into the juice. 

**Figure 2:**
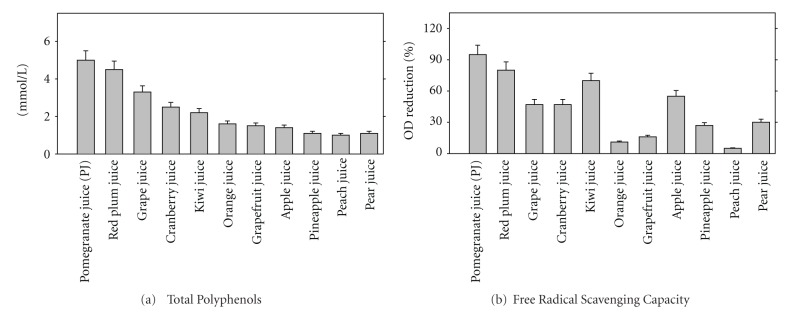
Fruit juices total polyphenols concentration and free radical scavenging capacities. Total polyphenol concentration of various juices was determined using quercetin as a standard (a). Free radical scavenging capacity was analyzed by the DPPH assay and is given as % of absorbance reduction by 1 *μ*L/mL of juice after 5 minutes of incubation. Results are given as mean ± S.D of three different experiments.

We have demonstrated that PJ contains a higher concentration of total polyphenols (5 mmol/L) in comparison to other fruit juices (orange, grapefruit, grape, cranberry, pear, pineapple, apple, and peach juices which contain only 1.3–4 mmol/L of total polyphenols, Figure 2(a)). Determination of free radicals scavenging capacities of various juices revealed that PJ was the most potent one, whereas orange juice, grapefruit juice, and peach juice demonstrated very low free radicals scavenging capacities (Figure 2(b)). The antioxidant potency of commonly consumed polyphenol-rich beverages in the USA was also compared [34]. Total polyphenol content in these beverages was evaluated by gallic acid equivalents determination. This study applied five tests of antioxidant potency: (1) trolox equivalent antioxidant capacity (TEAC), (2) total oxygen radical absorbance capacity (ORAC), (3) free radical scavenging capacity by 2, 2-diphenyl-1-picrylhydrazyl (DPPH), (4) ferric reducing antioxidant power (FRAP), and (5) inhibition of LDL oxidation. The beverages included several different brands as follows: apple juice, acai juice, black cherry juice, blueberry juice, cranberry juice, Concord grape juice, orange juice, red wines, iced tea beverages (black tea, green tea, white tea), and the major PJ available in the U.S. market. An overall antioxidant potency composite index was calculated by assigning each test equal weight. PJ had the greatest antioxidant potency composite index among the beverages tested, and was at least 20% greater than any of the other beverages tested. Antioxidant potency, ability to inhibit LDL oxidation, and total polyphenol content were consistent in classifying the antioxidant capacity of the polyphenol-rich beverages in the following order: PJ > red wine > Concord grape juice > blueberry juice > black cherry juice, acai juice, cranberry juice > orange juice, iced tea beverages, apple juice. Although *in vitro* antioxidant potency does not prove *in vivo* biological activity, there was a consistent clinical evidence of antioxidant potency for the most potent beverage antioxidants with PJ and red wine being most potent. Furthermore, we have recently compared the antioxidative properties of 35 beverages, and found that 100% Wonderful-variety pomegranate and 100% black currant juices were both, the most potent antioxidants *in vitro* and *in vivo*, as they inhibited copper ion-induced LDL oxidation by up to 94% and AAPH-induced serum lipid peroxidation by up to 38% [35].

The most potent antioxidant activity of PJ could be related to its high polyphenolic flavonoid content, as well as, to the specific type of potent polyphenols present in PJ (specific hydrolyzable tannins).

### 3.2. Contribution of PJ Constituents to Its Antioxidative Properties

Several polyphenolic fractions were isolated from PJ, including gallic acid, ellagic acid, tannins, total PJ anthocyanins, and specific anthocyanins, such as cyanidin-3-0-*β*-glucopyranoside, cyanidin-3,5-di-0-*β*-glucopyranoside, delphinidin-3-0-*β*-glucopyranoside, and pelargenin-3-0-*β*-glucopyranoside. The total anthocyanin and tannin fractions exhibited a dose-dependent antioxidative effect against copper ion-induced LDL oxidation. In the AAPH-induced LDL oxidation process, both fractions exhibited weaker antioxidative properties in comparison to the copper ion-induced LDL oxidation. These results suggest that the anthocyanins and tannins possess in addition to their free radical scavenging capabilities, also transition metal ion chelation properties. The tannin fraction was more potent than the anthocyanin fraction in inhibiting LDL oxidation, and the IC_50_ of the tannins was half that of the anthocyanins. Both PJ, ellagic and gallic acids and the anthocyanins delphinidin-3-0-*β*-glucopyranoside, pelargonidin-3-0-*β*-glucopyranoside, cyanidin-3-0-*β*-glucopyranodise, and cyanidin-3, 5-di-0-*β*-glucopyranoside inhibited copper ion-induced LDL oxidation in a dose-dependent manner. Upon comparing the effects of ellagic acid to gallic acid, gallic acid was more potent inhibitor of LDL oxidation (IC_50_ of 2.1 *μ*g/mL for gallic acid, versus 16 *μ*g/mL for ellagic acid). Similarly, the anthocyanins delphinidin-3-0-*β*-glucopyranoside and cyanidin-3-0-*β*-glucopyranodise were more potent antioxidants against LDL oxidation than ellagic acid, with IC_50_ of 3.0, 2.0, and 16 *μ*g/mL, respectively. When comparing the antioxidative properties of the specific PJ anthocyanins, pelargonidin-3-0-*β*-glucopyranoside and cyanidin-3, 5-di-0-*β*-glucopyranoside were less potent than delphinidin-3-0-*β*-glucopyranoside or cyanidin-3-0-*β*-glucopyranodise (IC_50_ of 13 *μ*g/mL versus 2-3 *μ*g/mL). A similar pattern was noted for the free radical scavenging capabilities of the above PJ fractions.

## 4. PJ Consumption Reduces Serum Oxidative ****Stress

### 4.1. Serum Lipid Peroxidation

Human plasma obtained from healthy subjects after 2 weeks of PJ consumption (50 mL PJ concentrate/day, equivalent to 1.5 mmol total polyphenols) demonstrated a small but significant (*P* < 0.01) 16% decreased susceptibility to free radical-induced lipid peroxidation, in comparison to plasma obtained prior to PJ consumption, as measured by lipid peroxides formation, or by total antioxidant status (TAS) in serum. To determine the effect of increasing or decreasing the dosages of PJ on plasma lipid peroxidation, and to analyze PJ capability to maintain its effect after termination of juice consumption, three subjects were further studied. Supplementation of 20 mL of PJ concentrate/day for one week resulted in a significant decrease of 11% in plasma lipid peroxidation, compared to plasma obtained prior to PJ consumption. Supplementation of 50 mL PJ concentrate/day for one more week exhibited a further 21% decrease in plasma lipid peroxidation. However, a further increase in the supplemented PJ to 80 mL of PJ concentrate/day for an additional one week did not further inhibit plasma susceptibility to lipid peroxidation. Gradual decreasing of the PJ dosage in these 3 subjects down to 40 mL/day for one week, and then to 20 mL/day for additional two weeks, did not significantly affect plasma lipid peroxidation, which remained low in comparison to the levels obtained after supplementation of 80 mL of PJ concentrate/day. Two weeks after cessation of PJ supplementation, the reduced rate of plasma susceptibility to lipid peroxidation was sustained. After a further 4 weeks with no PJ consumption, plasma lipid peroxidation returned to the higher values obtained before PJ consumption. 

**Figure 3:**
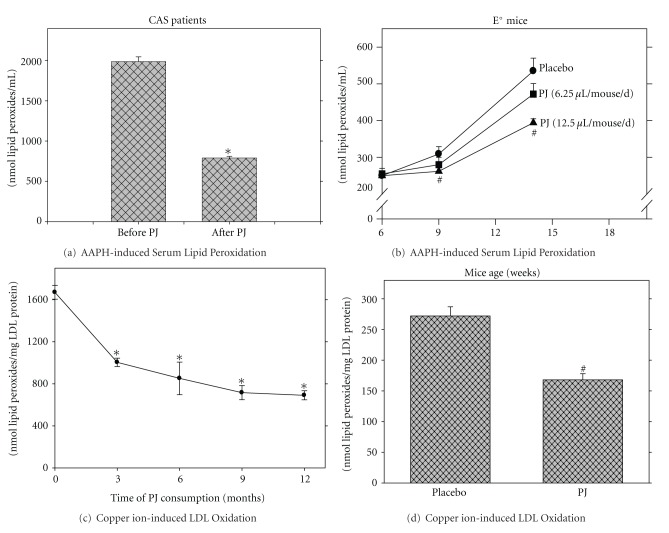
Pomegranate juice (PJ) consumption reduces serum or LDL oxidation in patients with carotid artery stenosis (CAS) and in the atherosclerotic E^0^ mice. Effect of PJ supplementation to CAS patients (for 1 year) or to E^0^ mice (for up to 14 weeks) on (a and b) the susceptibility of serum to radical-induced lipid peroxidation, and on (c and d) copper ion-induced LDL oxidation is shown.*P < 0.01 (mean ± SD, after PJ versus before PJ consumption in humans) and ^#^ *P* < 0.01 (mean  ±  SD, PJ (12.5 *μ*L/mouse/day) versus placebo in mice).

The effect of PJ consumption by patients with CAS on their serum oxidative state was also measured [32]. A significant (*P* < 0.01) reduction in the concentration of antibodies against Ox-LDL by 24% and 19% was observed after 1 and 3 months of PJ consumption, respectively, (from 2070 ± 61 EU/mL before treatment to 1563 ± 69 and 1670 ± 52 EU/mL after 1 and 3 months of PJ consumption, resp.). TAS in serum from these patients was substantially increased by 2.3 fold (from 0.95 ± 0.12 nmol/L at baseline, up to 2.20 ± 0.25 nmol/L after 12 months of PJ consumption). These results indicate that PJ administration to patients with CAS substantially reduced their serum oxidative status and could thus inhibit plasma lipid peroxidation. The susceptibility of the patient's serum to free radical-induced oxidation decreased after 12 months of PJ consumption by 62% (Figure 3(a)). The effect of PJ consumption on serum oxidative state was recently measured also in patients with noninsulin dependent type 2 diabetes mellitus. Consumption of 50 mL of PJ per day for a period of 3 months resulted in a significant reduction in serum lipid peroxides and TBARS levels by 56% and 28%, respectively [36].

PJ consumption exhibited antioxidative effects also when administered to E^0^ mice [28]. The basal oxidative state, measured as lipid peroxides in serum of control E^0^ mice (that did not consume PJ), increased gradually during aging from 260 nmol/mL of serum at 6 weeks of age, to 309 and 535 nmol/mL of serum after 9 and 14 weeks of age, respectively. Following PJ consumption, the extent of serum lipid peroxidation by the free radical AAPH was markedly reduced, as compared to that observed in serum from the placebo mice, and this effect was PJ concentration-dependent (Figure 3(b)). Similarly, serum TAS was higher in E^0^ mice that consumed PJ, in comparison to control mice, and this effect was again juice concentration-dependent [28]. 

### 4.2. Serum LDL and HDL Oxidation

Consumption of PJ for 1 and 2 weeks by healthy volunteers increased the resistance of their LDL to copper ions-induced oxidation, as shown by a prolongation of the lag time required for the initiation of LDL oxidation, by 29% and 43%, in comparison to LDL obtained prior to juice consumption [28]. Similarly, the resistance of their HDL to copper ion-induced oxidation also gradually increased after PJ consumption, as shown by a prolongation in the lag time required for the initiation of HDL oxidation from 37 ± 2 minutes to 45 ± 6 minutes before and 2 weeks after PJ consumption, respectively. 

PJ consumption by patients with CAS resulted in a significant reduction in the basal level of LDL-associated lipid peroxides by 43%, 89%, 86%, and 90% after 3, 6, 9, and 12 months of PJ consumption, respectively, and in parallel, it increased the resistance of LDL to copper ion-induced oxidation [32]. This was demonstrated by reduced formation of lipid peroxides in LDL during its incubation with copper-ions (by 40%, 49%, 57%, and 59% after 3, 6, 9, and 12 months of PJ consumption, respectively, Figure 3(c)). PJ consumption also decreased the propensity of LDL derived from E^0^ mice to copper ion-induced oxidation (Figure 3(d)). In E^0^ mice that consumed 6.25 *μ*L/mouse/d or 12.5 *μ*L/mouse/d of PJ concentrate for a period of 3.5 months, LDL oxidation by copper ions was delayed by 100 minutes and by 120 minutes, respectively, in comparison to LDL obtained before juice administration. Determination of the extent of LDL oxidation by the lipid peroxides assay revealed a significant inhibition after PJ consumption (Figure 3(d)). Furthermore, the progressive increase with age in the susceptibility of the mice LDL to oxidation was significantly attenuated by PJ consumption, in a dose-dependent manner [28].

### 4.3. Serum Paraoxonase 1 (PON1)

The increased resistance of LDL and of HDL to oxidation after PJ administration to healthy subjects or to patients with CAS could have also resulted from increased serum HDL-associated paraoxonase1 (PON1) activity. Indeed, a significant 18% increase in serum PON1 activity was monitored in healthy subjects after PJ consumption for a period of 2 weeks [28]. In CAS patients, serum PON1 arylesterase activity significantly increased by 11%, 42%, 49%, and 83% after 3, 6, 9, and 12 months of PJ consumption, respectively [32], and in patients with type 2 diabetes mellitus it significantly increased by 12% after PJ consumption for 3 months [36]. In another study from our group, we analyzed thirty patients with type 2 diabetes mellitus. Ten male patients and 10 female patients received concentrated Wonderful variety of PJ (WPJ, 50 mL/day for 4 weeks), while another group of 10 male patients received pomegranate by-product extract (WPOMxl, 5 mL/day for 6 weeks) [37]. There were no significant effects of WPJ or WPOMxl consumption on fasting blood glucose or hemoglobin A1c levels. After 4 weeks of WPJ consumption by male patients, basal serum oxidative stress was significantly decreased by 35%, whereas serum concentration of sulfhydryl (SH) groups (antioxidant marker) was significantly increased by 25%. In male patients that consumed WPOMxl and in female patients that consumed PJ, a similar pattern was observed, though to a lesser extent.

**Figure 4:**
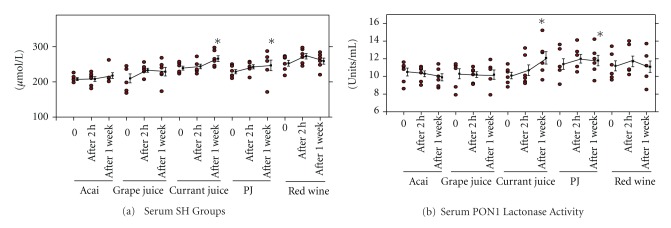
Pomegranate juice (PJ) or black currant juice consumption by healthy subjects, for a short term, decreases serum oxidative stress and increases PON1 activity. Six healthy subjects consumed 250 mL/day of pomegranate juice (PJ, POM Wonderful), black currant juice (Knudsen), Red Wine (Mondavi), Grape Juice (Lakewood), or Acai Juice (Naked) for up to one week, followed by one week of washout. Blood samples were collected before and after 2 hours or one week of beverages consumption. (a) Serum SH groups' concentration. (b) Serum paraoxonase 1 (PON1) lactonase activity (towards dihydrocoumarin) were determined. The individual results, as well as, the mean ± SD for each treatment at the different time points are shown. *P < 0.01 versus time 0.

The effect of consuming the polyphenol-richest beverages (Wonderful-variety pomegranate juice, black currant juice, Concord grape juice, acai juice blend, and red wine) by healthy subjects was further analyzed *in vivo* for a short term (after 2 hours, and after 1 week of consumption) [35]. Consumption of these antioxidant rich beverages (especially Wonderful-variety pomegranate juice and black currant juice) increased serum SH groups level already after 2 hours, and more so, after 1 week (Figure 4(a)). After 1 week of consumption, black currant juice or Wonderful-variety pomegranate juice significantly increased serum SH groups concentration by 11% or 8%, respectively (Figure 4(a)). In contrast, consumption of the other beverages for 2 hours or for one week had no statistically significant effect on serum SH groups' concentration (Figure 4(a)). We next analyzed the effects of selected polyphenols-rich beverages consumption by healthy subjects (2 hours or 1 week consumption) on serum PON1 catalytic activity (Figure 4(b)) [35]. Two hours after consumption of the selected beverages, serum PON1 lactonase activity was not significantly affected (Figure 4(b)). However, after 1 week of consumption, black currant juice significantly increased serum PON1 activities by 20%, and Wonderful-variety PJ significantly increased it by 5% (Figure 4(b)).

**Figure 5:**
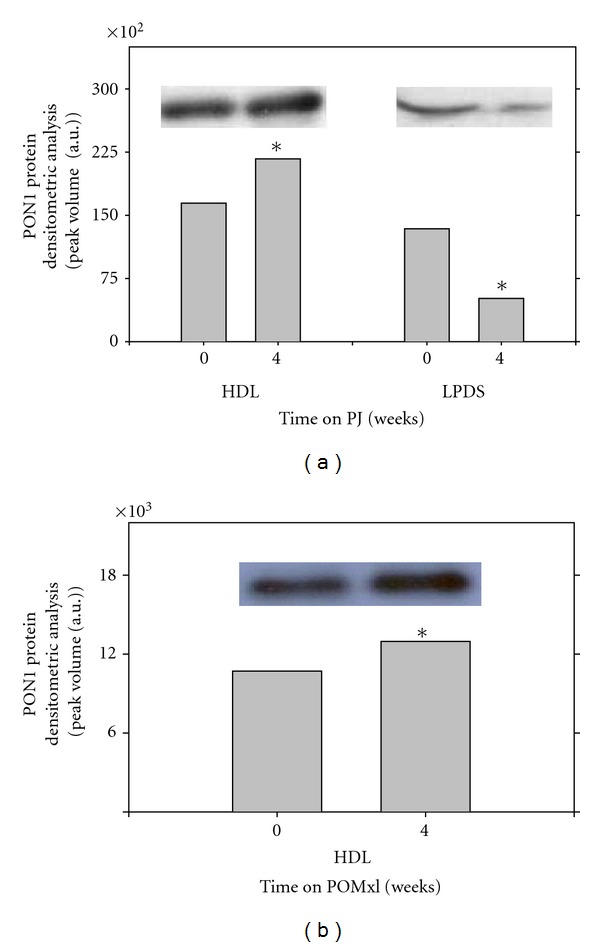
Pomegranate juice (PJ) or whole pomegranate fruit extract (POMxl) increase PON1 binding to HDL. Twenty patients with type 2 diabetes mellitus participated in the study. Ten male patients received concentrated PJ (50 mL/day for 4 weeks) while another group of 10 male patients received POMxl (5 mL/day for 6 weeks). (a) Blood samples were collected from both groups before (0 time) and 4 weeks after PJ consumption, or (b) 4 weeks after POMxl consumption. The HDL or LPDS fractions were isolated from the blood samples of 4 patients by density gradient ultracentrifugation. The HDL fractions (25 *μ*g protein) or LPDS fractions (20 *μ*L) were loaded on 10% acrylamide gel, and PON1 protein bands were visualized using mouse anti-human PON1 antibody. PON1 bands and densitometric analysis of the PON1 bands are shown. This is a representative experiment out of four. *P < 0.01 versus time 0.

Association of PON1 with HDL stabilizes the enzyme. In diabetic patients, PON1 dissociates from HDL and, as a consequence, it is less biologically active. We thus investigated the effects of PJ and POMxl consumption on PON1 association with HDL in diabetic patients [37]. HDL-associated PON1 arylesterase, paraoxonase, and lactonase activities increased significantly after PJ consumption, by 34–45%, as compared to the baseline levels. In male patients that consumed POMxl, and in female patients that consumed PJ, a similar pattern was observed, although to a lesser extent. PON1 protein binding to HDL was significantly increased by 32% following PJ consumption (Figure 5(a)), while the level of PON1 in the lipoprotein deficient serum (LPDS) decreased by 62% (Figure 5(a)), suggesting that PJ consumption resulted in increased free PON1 binding to the HDL. A similar trend of increased PON1 protein association with HDL was observed in males following POMxl consumption, as after 4 weeks of POMxl consumption, HDL-bound PON1 protein increased by 17%, as compared to baseline values (Figure 5(b)). The above results were confirmed also in *in vitro* study where serum from diabetic patients was incubated with PJ or with punicalagin, or with no addition (control) for 2 hours at 37°C. Then, HDL was isolated from the serum by ultracentrifugation, and Western blot analysis was performed. After serum incubation with PJ (18 *μ*g GAE/mL) or with punicalagin, the protein content of HDL-bound PON1 significantly increased by 36% and by 14%, respectively, as compared to control serum. Upon increasing the concentration of PJ or punicalagin up to 36 *μ*g GAE/mL, HDL-bound PON1 protein further increased, and it was 62% or 83% higher than that observed in control serum (no PJ), respectively [37]. 

We thus conclude that PJ, as well as, POMxl consumption by diabetic patients contributes to PON1 stabilization, by increasing its association with HDL, and therefore, enhancing PON1 catalytic activities. The ratio between HDL-associated PON1 and free PON1 gradually decreased as the extent of HDL oxidation increased. The antioxidants vitamin E or PJ inhibited the oxidation-mediated redistribution of PON1 in serum. Indeed, PJ or its purified major polyphenols punicalagin, gallic acid, or ellagic acid, all increased PON1 binding also to HDL. Furthermore, PON1 associated more efficiently with HDLs isolated from diabetic patients after PJ consumption versus patient HDLs isolated prior to PJ consumption [38].

**Figure 6:**
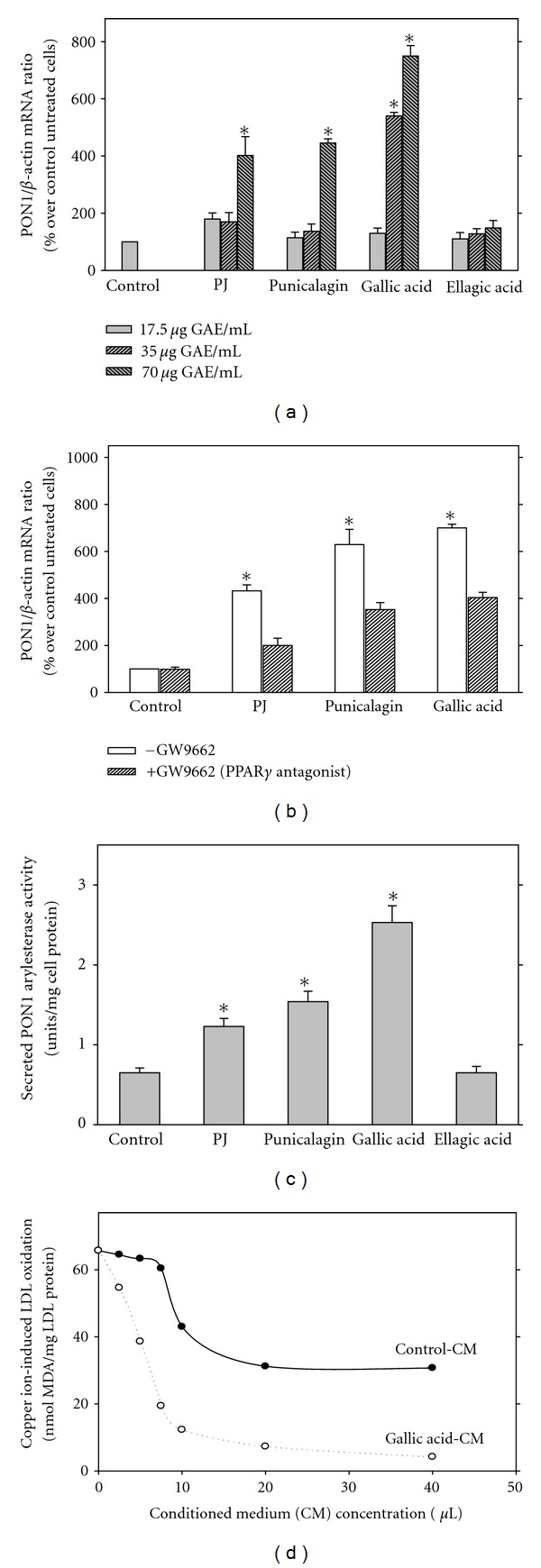
Pomegranate Juice (PJ) polyphenols upregulated paraoxonase 1 (PON1) expression in hepatocytes, via PPAR*γ*, resulting in the secretion of biologically active PON1. (a) PON1 mRNA expression in human hepatoma cell line (HuH7) that were incubated with increasing concentrations (17–70 *μ*g of gallic acid equivalents (GAE)/mL) of PJ, or its polyphenols: punicalagin, gallic, or ellagic acids, for 24 hours at 37°C. (b) PON1 mRNA expression in HuH7 cells, untreated (Control) or treated with pomegranate or its major polyphenols, in the absence or presence of the PPAR*γ* antagonist GW9662 (50 *μ*mol/L). (c) Secreted PON1 arylesterase activity was measured in the medium from control cells, or from pomegranate polyphenols-treated cells in the presence of HDL from PON1 knockout (KO) mice. (d) LDL was oxidized by copper ions in presence of conditioned medium from untreated (Control-CM) and from gallic acid treated cells (Gallic acid-CM). Results are expressed as mean ± SD of three different experiments. *P < 0.01 versus control.

Similarly to the results obtained in humans, a significant 43% increase in serum PON1 activity was also observed in E^0^ mice after PJ consumption for a period of 2 months, in comparison to serum PON1 activity observed in the placebo-treated mice [29]. The increase in serum PON1 activity may be a direct effect of PJ, as well as an effect secondary to PJ-mediated reduction in lipid peroxides, as it was previously demonstrated that paraoxonase is inactivated by oxidized lipids [39], and its activity is preserved by antioxidants, such as PJ, red wine, or the licorice root-derived isoflavan glabridin. The above increment in PON1 catalytic activities could have resulted also from PJ-induced increment in liver PON1 expression. Indeed, PON1 protein and mRNA expression, as well as PON1 gene promoter activation, were significantly increased in hepatocytes cell line (HuH7) following incubation with PJ or its major polyphenols punicalagin, or gallic acid (GA), and this effect was dose-dependent (Figure 6(a)) [40]. This effect of PJ polyphenols was mediated, at least in part, via the transcription factor PPAR*γ*, as addition of PJ polyphenols to HuH7 cells in the presence of the PPARgamma antagonist GW9662, significantly decreased the stimulatory effect of PJ polyphenols on PON1 expression (Figure 6(b)) [40]. 

In accordance with the above increased PON1 mRNA expression, PJ, punicalagin, and GA increased the hepatocytes-secreted PON1 activity (in the presence of HDL from PON1KO mice) by 2.7 and 1.9 fold, respectively, compared to control untreated cells (Figure 6(c)). Functionally, the secreted PON1 exhibited biological activity, as it protected LDL from copper ion-induced oxidation (Figure 6(d)) [40].

## 5. PJ Reduces Macrophage Atherogenicity 

Oxidative stress has been implicated in the pathogenesis of atherosclerosis [41, 42], leading to the oxidation of lipids, not only in LDL, but also in arterial macrophages [43, 44]. We have previously shown that “lipid-peroxidized macrophages” exhibit atherogenic characteristics, including increased ability to oxidize LDL and to take up oxidized LDL (Ox-LDL) [45].

We thus studied the effect of dietary consumption of PJ by E^0^ mice on macrophage atherogenicity, including macrophage lipid peroxidation and subsequently macrophage activities related to foam cell formation, such as cell-mediated oxidation of LDL and cellular uptake of lipoproteins.

### 5.1. Macrophage Oxidative Stress

**Figure 7:**
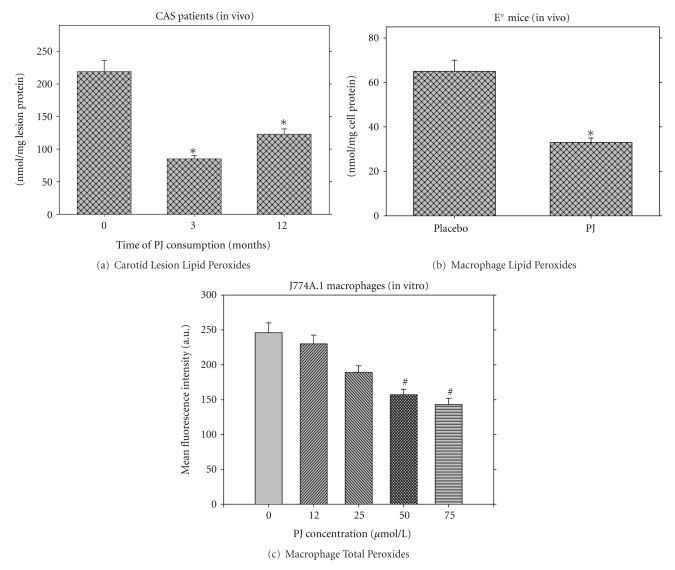
Pomegranate juice (PJ) reduces oxidative stress in carotid artery stenosis (CAS) patients' lesion, and in macrophages. Effect of PJ consumption by CAS patients on lipid peroxides content in human carotid lesions (a), and the effect of PJ consumption by E^0^ mice on lipid peroxides content in their mouse peritoneal macrophages (b). J774A.1 macrophage cell line was incubated with increasing concentrations of PJ for 20 hours at 37°C, followed by cellular oxidative stress analysis measured as DCFH oxidation (c).*P < 0.01 (mean±SD versus 0 time in humans, and PJ versus placebo in the mice study), ^#^ *P* < 0.01 cell incubation with PJ versus control cells (without PJ).

We have demonstrated that human monocyte derived macrophages (HMDM) isolated from patients with type 2 diabetes mellitus after consumption of PJ for 3 months, as well as, the carotid lesion derived after endarterectomy from CAS patients that consumed PJ (Figure 7(a)), and also mouse peritoneal macrophages (MPM) isolated from E^0^ mice after consumption of PJ concentrate (12.5 *μ*L/mouse/day, equivalent to 0.35 *μ*moles of total polyphenols) for a period of 3.5 months (Figure 7(b)), contained less lipid peroxides, in comparison to carotid lesion from patients that did not consume PJ, or to MPM from the placebo-treated E^0^ mice, respectively [28, 32]. Incubation of the human carotid lesion or of E^0^ MPM with LDL (100 *μ*g of protein/mL) for 18 hours under oxidative stress (in the presence of copper ions) revealed that PJ consumption resulted in 43% and 82% reduced capacity of the lesion or the macrophages to oxidize LDL, respectively. The mechanism responsible for this effect was associated with inhibition of the translocation to the macrophage plasma membrane of the NADPH oxidase cytosolic factor p-47, and hence, inhibition of NADPH oxidase activation and of superoxide anion release from the macrophages.

Similarly to the *in vivo* studies, preincubation of J774A.1 macrophages with increasing concentrations of PJ dose-dependently reduced macrophage oxidative stress, as measured by total peroxides level (Figure 7(c)). Recently, we have demonstrated that unique complex sugars and/or phenolics in PJ also contribute to PJ-induced reduction of macrophage oxidative stress [46]. Increasing concentrations of the PJ sugar fractions resulted in a dose-dependent decrement in J774A.1 macrophage cell line peroxide levels, up to 72%, compared to control cells. On the contrary, incubation of the cells with white grape juice sugar fraction at the same concentration resulted in a dose-dependent increment in peroxide levels up to 37%. On the molecular basis, PJ-induced reduction in macrophage oxidative stress could result from the effect of PJ on the transcription of redox sensitive genes. The activation of the oxidative stress responsive transcription factor, Nuclear Factor kappa-B (NF*κ*-B), has been linked with a variety of inflammatory diseases, including atherosclerosis. Extensive research in the last few years, reviewed by Aggarwal and Shishodia [47], has shown that the pathway that activates NF*κ*-B can be inhibited by phytochemicals, including those present in pomegranate, thus providing a beneficial effect against atherosclerosis development. It was demonstrated that pomegranate wine (PJ fermented with yeast and dealcoholized) inhibits oxidation of endothelial cells induced by TNF-*α*, and acts as a potent inhibitor of NF*κ*-B activation in these cells [48]. Pomegranate fermented juice and pomegranate cold pressed seed oil flavonoids were also shown to inhibit eicosanoid enzyme activity [49]. Flavonoids extracted from pomegranate cold pressed seed oil showed 31–44% inhibition of sheep cyclooxygenase and 69–81% inhibition of soybean lipoxygenase. Flavonoids extracted from pomegranate fermented juice also showed 21–30% inhibition of soybean lipoxygenase. Recently, it was demonstrated that PJ decreased the activation of the redox-sensitive genes ELK-1 and p-JUN and increased eNOS expression in cultured endothelial cells which were exposed to shear stress, as well as in atherosclerotic-prone areas of hypercholesterolemic mice [30]. 

PJ-mediated reduction in the transcription of several key redox enzymes, including cyclooxygenase, lipoxygenase, and NO synthase could be the result of intracellular oxidation suppression. Through this mechanism, as well as via the suppression of lipoxygenase-catalyzed leukotriene formation, PJ may act also as an anti-inflammatory agent, in addition to its major role as antioxidant.

### 5.2. Macrophage Paraoxonase 2 (PON2)

**Figure 8:**
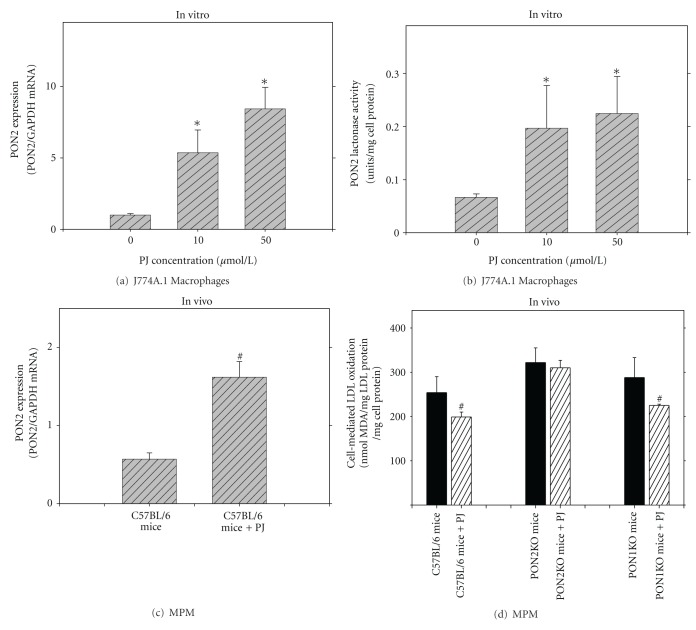
Pomegranate juice (PJ) upregulates macrophage paraoxonase 2 (PON2) expression and reduces macrophage oxidative stress. (a) and (b) *In vitro* studies. J774A.1 macrophages were incubated with increasing concentrations (0–50 *μ*mol polyphenols/L) of PJ for 20 hours. Then, PON2 mRNA expression (a), as well as PON2 lactonase activity towards dihydrocoumarin (b) were determined. (c) and (d) *In vivo* studies. C57BL/6 mice, as well as PON1knockout (KO) mice, or PON2KO mice, consumed PJ (200 *μ*g of gallic acid equivalents/mouse/day) for 1 month, and the mice peritoneal macrophages (MPM) were harvested. MPM PON2 mRNA expression (c) and the cells' ability to oxidize LDL in the presence of copper ions were measured (d). *P < 0.01 (incubation *in vitro* with PJ versus without PJ), ^#^ *P* < 0.01 (after PJ versus before PJ consumption).

Whereas PON1 is expressed mainly in the liver and is present in the circulation, PON2 is expressed in most tissues, including macrophages, but it is not present in the circulation [50, 51]. PON2, like PON1 however, was shown to protect against atherosclerosis development [52]. This effect could be attributed to PON2-induced reduction in macrophage oxidative stress [50, 51, 53], as well as, in triglyceride accumulation [53, 54]. We next questioned the association between PJ polyphenolics, macrophage oxidative stress, and cellular PON2 expression, in relation to the activation of specific PON2 transcription factors [55]. Incubation of J774A.1 macrophages with PJ (0–50 *μ*mol/L of total polyphenols) dose-dependently increased PON2 mRNA (Figure 8(a)), protein expression, and lactonase activity (Figure 8(b)) and reduced macrophage oxidative stress. These effects could be attributed to the PJ unique polyphenols, punicalagin, and gallic acid. PJ polyphenol-induced upregulation of PON2 was inhibited by 40% upon using the PPAR*γ* antagonist GW9662. Accordingly, the PPAR*γ* ligand, rosiglitazone, dose-dependently stimulated macrophage PON2 expression by up to 80%. Inhibition of AP-1 activation (with SP600125) attenuated PJ-induced upregulation of PON2 by 40%. Similarly, incubation of macrophages with PJ polyphenols in the presence of GW9662 or SP600125 significantly reduced their capacity to protect macrophages against oxidative stress [55]. We thus conclude that the antioxidative characteristics of PJ unique phenolics punicalagin and gallic acid could be related, at least in part, to their stimulatory effect on macrophage PON2 expression, a phenomenon which was shown to be associated with activation of the transcription factors PAPR*γ* and AP-1.We next analyzed the role of PON2 in the antiatherogenic properties of PJ. Consumption of PJ by C57BL/6 mice (Figure 8(c)), or by PON1KO mice significantly upregulated PON2 expression in MPM [56]. Unlike the inhibitory effects of PJ consumption in C576BL/6 MPM or in PON1KO MPM on macrophage oxidative stress (as measured by the cells' ability to oxidize LDL), in PON2KO mice, PJ consumption had a nonsignificant effect, on LDL oxidation rate by the cells (Figure 8(d)). These results indicate that PJ-induced reduction in macrophage oxidative stress is mediated mostly by PJ-induced macrophage PON2 (but not PON1) overexpression. 

### 5.3. Macrophage Cholesterol Metabolism

Macrophage cholesterol accumulation and foam cell formation are the hallmark of early atherogenesis. Cholesterol accumulation in macrophages can result from impaired balance between external and internal cholesterol sources. LDL, which undergoes oxidative modification, is an important external source for macrophage accumulated cholesterol. 

Ox-LDL is taken up by macrophages at enhanced rate via scavenger receptors [57, 58], which, unlike the LDL receptor, are not down regulated by intracellular cholesterol content [59], and therefore lead to accumulation of cholesterol in the cells. Macrophage cholesterol from internal sources origins from cholesterol biosynthesis. The enzyme 3-hydroxy-3 methylglutaryl coenzyme A (HMGCoA) reductase catalyzes the rate limiting step in cholesterol biosynthetic pathway [60], and it is subjected to a negative feedback regulation by the cellular cholesterol content. In addition to cellular uptake of lipoproteins and to cholesterol biosynthesis, macrophage cholesterol accumulation can also result from a decreased efflux of cholesterol from the cells [61]. 

Since PJ was shown to inhibit macrophage foam cell formation and the development of atherosclerotic lesions, we analyzed the effect of PJ consumption on cellular processes that lead to macrophage cholesterol accumulation. We have demonstrated that the cellular uptake of Ox-LDL, measured as cellular lipoprotein binding, cell-association, and degradation, by MPM derived from E^0^ mice that consumed 12.5 *μ*L of PJ concentrate/mouse/day for a period of 2 months, was significantly reduced, by 16%, 22%, and 15%, respectively, in comparison to Ox-LDL binding, cell-association, and degradation obtained by MPM from control E^0^ mice. Similarly, PJ consumption by patients with type 2 diabetes mellitus significantly decreased the extent of Ox-LDL cellular uptake by their HMDM (by 36%) [36]. Cellular cholesterol esterification rate (another atherogenic property of macrophages) in MPM isolated from PJ-treated mice was found to be 80% lower compared with age-matched, placebo-treated mice. Finally, PJ treatment significantly increased, by 39%, cholesterol efflux from macrophages compared with the cholesterol efflux rate from MPM harvested from the placebo-treated mice. Taken together, all these antiatherogenic effects lead to reduced accumulation of cholesterol in macrophages.

*In vitro* studies clearly show that PJ exhibit direct antiatherogenic effects on macrophages. Preincubation of J774A.1 macrophages with PJ resulted in a significant (*P* < 0.01) reduction in Ox-LDL degradation by 40% [62]. On the contrary, PJ had no effect on macrophage degradation of native LDL and also not on macrophage cholesterol efflux. Macrophage cholesterol biosynthesis however, was inhibited by 50% after cell incubation with PJ. This inhibition, unlike statin action, was not mediated by an effect on HMGCoA reductase along the cholesterol biosynthetic pathway. 

### 5.4. Macrophage Triglyceride Metabolism

**Figure 9:**
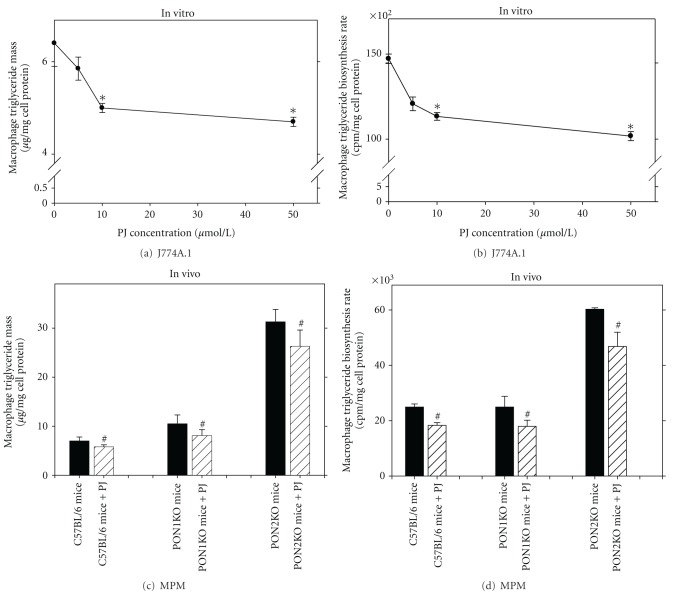
Pomegranate juice (PJ) protects macrophages from triglyceride accumulation: *in vitro* and *in vivo* studies. (a) and (b) *In vitro* studies: J774A.1 macrophages were incubated with increasing concentrations (0–50 *μ*mol polyphenols/L) of PJ, for 20 hours. Cellular triglyceride content (a) and macrophage triglyceride biosynthesis rate (b) were determined. (c) and (d) *In vivo* studies: C57BL/6 mice, as well as PON1knockout (KO) mice, or PON2KO mice, consumed PJ (200 *μ*g of gallic acid equivalents (GAE)/mouse/day) for 1 month, and the mice peritoneal macrophages (MPM) were then harvested. Cellular triglyceride content (c) and macrophage triglyceride biosynthesis rate (d) were determined. *P < 0.01 (incubation *in vitro* with PJ versus without PJ), ^#^ *P* < 0.01 (after PJ versus before PJ consumption).

Triglycerides are an independent risk factor for atherosclerosis [63]. Macrophage foam cells isolated from atherosclerotic lesions contain not only cholesteryl esters and unesterified cholesterol, but also substantial amount of triglycerides [64]. Triglyceride accumulation in macrophages increases oxidative stress and cellular necrosis, thus further contributing to foam cell formation [65]. The effect of PJ on macrophage triglyceride metabolism was recently studied [66]. Upon incubation of J774A.1 macrophages with PJ (0–50 *μ*M), a dose-dependently decrease (by up to 30%) in cellular triglyceride content (Figure 9(a)), and in triglyceride biosynthesis rate (Figure 9(b)) were observed. Similarly, punicalagin, the major PJ polyphenol, inhibited MPM triglyceride biosynthesis rate by 40%. Triglyceride hydrolytic rate however was not significantly affected by PJ or punicalagin. The activity of diacylglycerol acyltransferase 1 (DGAT1, the rate limiting enzyme in triglycerides biosynthesis) was significantly inhibited, (by ~50%), in J774A.1 macrophages that were treated with 50 *μ*M of PJ or with punicalagin, with no significant effect on DGAT1 mRNA or protein expression. Both PJ and punicalagin increased (by ~1.7 fold) MPM PON2 mRNA expression, and PON2 was previously shown to inhibit DGAT1 activity [53]. Addition of PJ or punicalagin (50 *μ*mol/L) to microsomes from PON2KO MPM still resulted in a significant reduction in DGAT1 activity, by ~50%, suggesting a direct, non-PON2-mediated effect of PJ on macrophage triglycerides [66]. Similarly to the *in vitro* study, we have recently demonstrated that PJ consumption by C57BL/6 mice, by PON1KO mice, or by PON2KO mice resulted in a significant reduction in MPM triglyceride content by 17%, 16%, and 23%, respectively (Figure 9(c)), and this effect was associated with a significant reduction in macrophage triglyceride biosynthesis rate by 27%, 28%, or 22%, respectively (Figure 9(d)) [56].

 This study demonstrates that PJ beneficial effects on macrophage triglyceride metabolism are direct effect of PJ, which are not mediated via PJ-induced stimulation of macrophage PON2.

We conclude that PJ protects the macrophages against oxidative stress by increasing PON2 expression, suppressing Ox-LDL uptake by macrophages, inhibiting cellular cholesterol and triglyceride biosynthesis, or stimulation of HDL-mediated cholesterol efflux from the cells. All these effects could lead to attenuation in cellular cholesterol and triglyceride accumulation, and foam cell formation.

## 6. Antiatherogenic Effects of Pomegranate ****By-Products

Pomegranate extract prepared from the whole fruit contains an approximately 20-fold increased antioxidant activity in comparison to the level in the pomegranate aril juice [67]. The antioxidant level in the pomegranate whole extractis directly correlated with the content of hydrolyzable tannins (in which punicalagin is predominant), while no such correlation was found for the level of anthocyanins [67]. 

A product of total pomegranate tannins (TPT) containing 85% punicalagin, 1.3% ellagic acid, and a small amount of ellagic acid glycosides was purified from the pomegranate husk extract prepared by Dr. Navindra P. Seeram from the laboratory of Dr. David Heber at UCLA, USA [68]. On a similar polyphenol weight basis (10 *μ*g/mL), TPT was more potent than vitamin E or PJ when analyzed as a free radical scavenger. TPT reduces the absorbance in the DPPH solution by 75% in comparison to 62% and 48% reduction obtained by a similar total polyphenol content of PJ or by vitamin E, respectively. LDL oxidation induced either by copper ions or AAPH, was dose-dependently inhibited by TPT, with an IC_50_ of 2.1 and 1.4 *μ*g/mL, respectively. Macrophage oxidative status was also substantially decreased by 50% on using 80 *μ*mol/mL of TPT polyphenols. At a similar concentration, punicalagin was found to be even more potent than TPT in all the above assays.

The antioxidative properties of pomegranate polyphenol extract powder, as well as of pomegranate fiber powder were also analyzed. The pomegranate fiber powder polyphenol concentration was 8-fold lower, as compared to the polyphenols in the pomegranate extract powder (200 ± 6 nmol/mg versus 1580 ± 138 nmol/mg, resp.). The pomegranate extract was significantly more potent than the fiber powder in both scavenging of free radicals and in reducing macrophage oxidative stress. 

**Figure 10:**
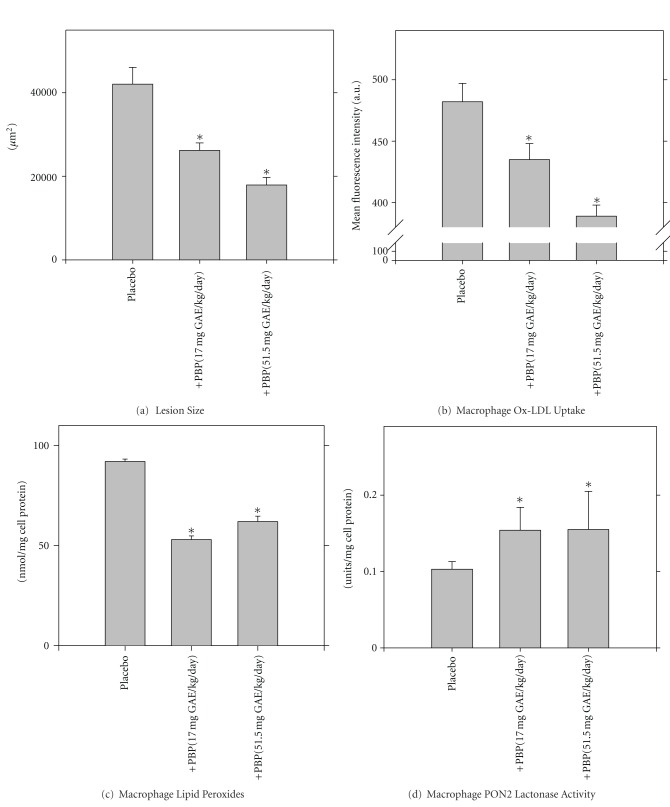
Pomegranate by-product (PBP) consumption by E^0^ mice attenuates atherosclerotic lesion development, in association with reduction in Ox-LDL uptake, and in macrophage oxidative stress, and on elevation in cellular paraoxonase 2 (PON2) activity. E^0^ mice consumed PBP (17 or 51.5 mg gallic acid equivalents (GAE)/kg/day) for 3 months. Control mice received only water (placebo). At the end of the study, the mice aortas, as well as, the mice peritoneal macrophages were harvested. (a) Atherosclerotic lesion size determination. (b) The extent of Ox-LDL (25 *μ*g of protein/mL, labeled with FITC) uptake by the mice macrophages was determined using flow cytometry. (c) Macrophage lipid peroxides level. (d) Macrophage PON2 lactonase activity towards dihydrocoumarin. Results are expressed as mean ± S.D of three different determinations. *P < 0.01 versus Placebo.

The effects of a pomegranate liquid by-product (PBP, which includes the whole pomegranate fruit left over after juice preparation), on atherosclerosis development in E^0^ mice were studied [69]. Consumption of PBP (17 or 51.5 mg gallic acid equivalents/kg/day) by the mice resulted in a significant reduction in atherosclerotic lesion size by up to 57% (Figure 10(a)). PBP consumption significantly reduced the extent of Ox-LDL uptake by the mice MPM by up to 19% (Figure 10(b)). Furthermore, MPM lipid peroxide content decreased by up to 42% (Figure 10(c)), after PBP consumption, in association with increment in PON2 lactonase activity by up to 50% (Figure 10(d)), as compared to MPM from the placebo mice. Similar results were observed also *in vitro*. Treatment of J774A.1 macrophages with PBP (10 *μ*mol/L or 50 *μ*mol/L of total polyphenols), significantly decreased both cellular total peroxide content and Ox-LDL uptake. We thus conclude that PBP significantly attenuates atherosclerosis development by its antioxidant properties. 

**Figure 11:**
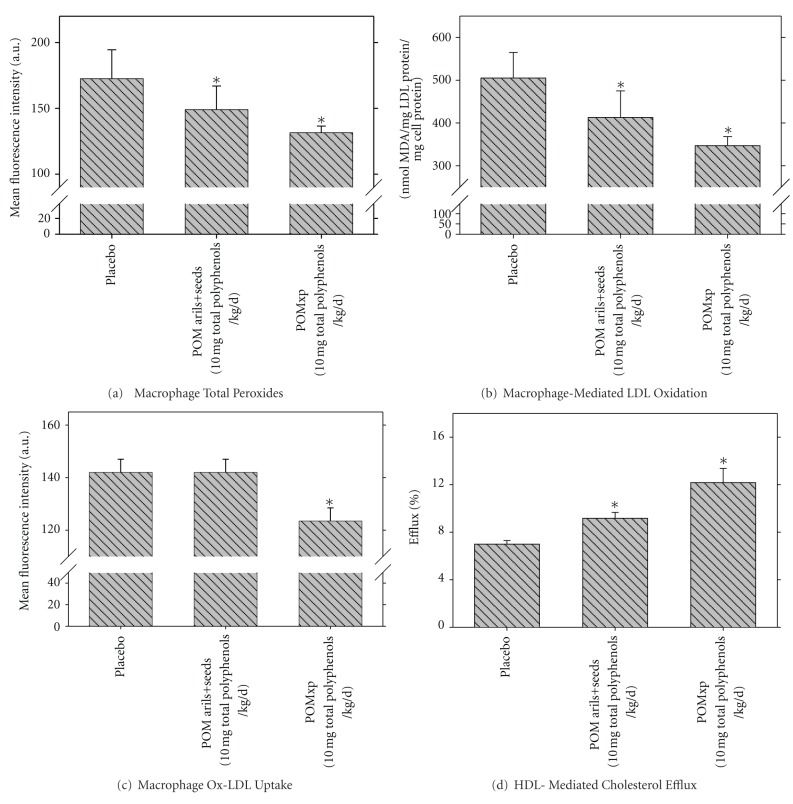
Increased *in vivo* antiatherogenic properties of whole pomegranate powder by-product (POMxp) versus pomegranate arils+seeds by-product (POM arils+seeds): studies in peritoneal macrophages from the atherosclerotic E^0^ mice. E^0^ mice (6 weeks old) consumed for 2 months 10 mg total polyphenols/kg/day of POMxp (dissolved in water), or of POM arils+seeds. Control mice received only water (placebo). At the end of the study, the mice peritoneal macrophages (MPM) were harvested. (a) Macrophage total peroxides content was determined by the DCFH assay. (b) The cells were incubated with LDL (100 *μ*g protein/mL) in the presence of copper ions, and the extent of LDL oxidation was measured by the TBARS assay. (c) The extent of Ox-LDL (25 *μ*g of protein/mL, labeled with FITC) uptake by the mice macrophages was determined using flow cytometry. (d) The mice macrophages were prelabeled with [^3^H]-cholesterol for 1 hour. Then, the cells were washed, and further incubated for 3 hours at 37°C without or with HDL (100 *μ*g of protein/mL). The extent of HDL-mediated cholesterol efflux was determined. Results are given as mean ± S.D of three different determinations. *P < 0.01 versus placebo.

Next, we performed *ex vivo* and *in vitro* studies, in which we compared the antiatherogenic properties of whole pomegranate fruit powder by- product (POMxp) to those of pomegranate by-product POMarils+seeds powder. Per mg powder, the POMxp contained 90-fold higher concentrations of polyphenols than the POMarils+seeds. Administration of 10 mg total polyphenols/kg/d of POMxp or of POMarils+seeds aqueous extract to E^0^ mice for 2 months, demonstrated that POMxp polyphenols are more potent than POMarils+seeds polyphenols in reducing oxidative stress. The amount of total peroxides in MPM from the mice that consumed POMarils+seeds, or POMxp was decreased by 14% or by 24%, respectively, as compared to MPM from placebo-treated mice (Figure 11(a)). Furthermore, the amount of superoxide anion released from MPM derived from E^0^ mice that consumed POMxp or POMarils+seeds was lower by 39% or by 14%, respectively, as compared to placebo MPM. This effect was associated with inhibition of MPM-mediated LDL oxidation by 18% or 31%, respectively (Figure 11(b)).

The extent of Ox-LDL uptake by MPM from mice that consumed POMxp, but not by MPM from the mice that consumed POMarils+seeds, was significantly reduced by 13%, as compared to Ox-LDL uptake by the placebo MPM (Figure 11(c)). Furthermore, consumption of POMxp or of POMarils+seeds by the E^0^ mice significantly stimulated HDL-mediated cholesterol efflux from the mice MPM by 74% and by 31%, respectively, (Figure 11(d)), indicating that the polyphenols in POMxp are more potent than the polyphenols in the by-product prepared from the arils+seeds in attenuation of macrophage cholesterol accumulation and foam cell formation. Similar results were observed *in vitro* upon adding POMxp or POMarils+seeds (10 mg gallic acid equivalents/mL) to J774A.1 macrophages, with 53% or 27% reduction in Ox-LDL uptake by the cells, respectively. Upon adding 41 *μ*g of gallic acid equivalents/mL of POMxp or POMarils+seeds to the cells, HDL-mediated cholesterol efflux was significantly stimulated by 147% and by 52%, respectively. 

**Figure 12:**
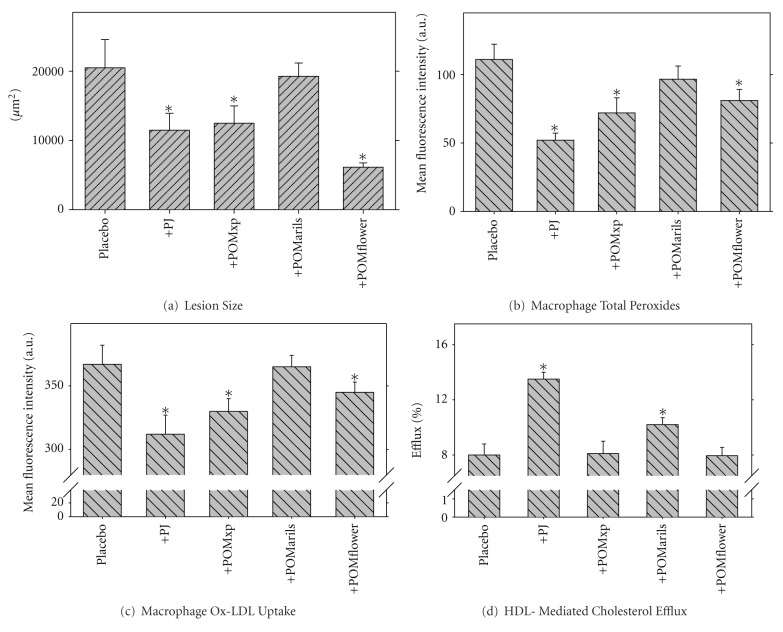
The effect of pomegranate extracts consumption by E^0^ mice on atherosclerotic lesion development and on the mice peritoneal macrophages atherogenicity. Fifty male E^0^ mice (6 weeks old) were divided into five groups of 10 mice each. The placebo group received only water. The other four groups received in their drinking water 200 *μ*g gallic acid equivalents (GAE)/mouse/day of: PJ, POMxp, POMarils, or POMflower for a 3-month period. At the end of pomegranate extracts consumption, the mice peritoneal macrophages (MPM), as well as the mice aortas were harvested. (a) Atherosclerotic lesion size in the mice aortas. (b) Macrophage total peroxides content was determined by the DCFH assay. (c) The extent of Ox-LDL (25 *μ*g of protein/mL, labeled with FITC) uptake by the mice macrophages was determined using flow cytometry. (d) The mice macrophages were prelabeled with [^3^H]-cholesterol for 1 hour. Then, the cells were washed and further incubated for 3 hours at 37°C without or with HDL (100 *μ*g of protein/mL). The extent of HDL-mediated cholesterol efflux was determined. Results are given as mean ± S.D of three different determinations. *P < 0.01 versus Placebo.

We next analyzed the antiatherogenic properties of the pomegranate fruit various parts including: peels (POMxp), arils (POMarils), and flowers (POMflower), in comparison to the whole pomegranate juice (PJ) [70]. After consumption of PJ, POMxp, or POMflower by the atherosclerotic E^0^ mice (200 *μ*g of GAE/mouse/day, for 3 months), the atherosclerotic lesion area was significantly decreased by 44, 39, or by as much as 70%, respectively, as compared to lesion area observed in the placebo-treated group (Figure 12(a)). POMarils consumption however, attenuated the lesion size by only 6% (Figure 12(a)). POMflower consumption reduced serum lipids and glucose levels by 18–25%. PJ, POMxp, POMflower, or POMarils consumption by the E^0^ mice resulted in a significant decrement by 53, 35, 27, or 13%, respectively, in MPM total peroxides content (Figure 12(b)) and significantly increased cellular PON2 activity, as compared to placebo-treated mice. The cellular uptake rates of Ox-LDL by E^0^ MPM (Figure 12(c)) were significantly reduced following the consumption of PJ (by 15%), or POMxp (by 10%). PJ and to a lesser extent POMarils consumption significantly stimulated HDL-mediated cholesterol efflux from MPM by 69% or by 28%, respectively (Figure 12(d)), whereas POMxp or POMflower consumption had no significant effect. We thus conclude that attenuation of atherosclerosis development by some of the pomegranate extracts and in particular POMflower could be related to their combined beneficial effects on macrophage atherogenic properties.

## 7. Perspectives and Future Directions

**Figure 13:**
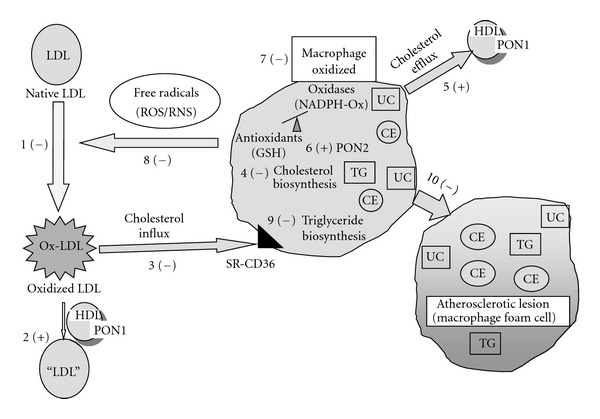
Major pathways by which pomegranate polyphenols inhibit macrophage foam cell formation and atherosclerosis development. Pomegranate polyphenols are the most potent antioxidants, and they directly inhibit LDL oxidation (#1). Pomegranate increases paraoxonase1 (PON1) liver expression and serum activity, thereby increasing hydrolysis of already formed lipid peroxides in oxidized LDL (Ox-LDL) (#2). Pomegranate polyphenols protect macrophages from cholesterol accumulation by several mechanisms: decrement in cholesterol influx, by inhibition of Ox-LDL uptake via the cells scavenger receptor CD36 (#3), or by inhibition of cholesterol biosynthesis rate (#4), or by stimulation of HDL-mediated cholesterol efflux from macrophages (#5). PJ polyphenols upregulate macrophage paraoxonase 2 (PON2) expression (#6), leading to reduction in macrophage oxidative stress (#7), and inhibition of reactive oxygen species (ROS), or reactive nitrogen species (RNS) production and release from the cells, which can lead to inhibition of LDL oxidation by the cells (#8). Pomegranate polyphenols also inhibit macrophage triglyceride (TG) biosynthesis rate (#9), thus reducing also TG accumulation in macrophages. Altogether, these antiatherogenic effects of PJ attenuate macrophage conversion into foam cells, and the development of atherosclerotic lesion (#10). CE, cholesterol ester; UC, unesterified cholesterol; TG, triglycerides, SR, scavenger receptor, (+), stimulation; (−), inhibition.

Our current view on the major pathways by which pomegranate polyphenols and pomegranate by-products reduce macrophage foam cell formation and the development of advanced atherosclerosis is summarized in Figure 13. Pomegranate fruit polyphenols (either in PJ or in the pomegranate by-products) protect against lipid peroxidation in serum, by direct interaction of pomegranate polyphenols with LDL, or indirect by increasing serum PON1 stability (HDL-association) and its catalytic activities, resulting in the hydrolysis of serum lipid peroxides. Moreover, PJ has a remarkable effect on macrophage atherogenicity. Pomegranate polyphenols were shown to reduce macrophage oxidative stress, secondary to pomegranate polyphenols-induced upregulation of macrophage PON2. PON2 inhibits the formation and release of reactive oxygen species (ROS) and reactive nitrogen species (RNS) in macrophages, thus preventing macrophage oxidation and the oxidation of LDL by the cells. 

PJ was shown to attenuate the accumulation of cholesterol in macrophages due to: (a) inhibition of cellular cholesterol biosynthesis, (b) inhibition of cellular Ox-LDL uptake, and (c) stimulation of HDL-mediated cholesterol efflux from macrophages. Furthermore, PJ also protects macrophages from triglyceride accumulation. Like PJ, pomegranate by-products were similarly shown (both *ex vivo* and *in vitro*) to reduce macrophage oxidative stress and to attenuate macrophage cholesterol accumulation and atherosclerotic lesion development.

All these antioxidative and antiatherogenic effects of pomegranate polyphenols were clearly demonstrated *in vitro*, as well as *in vivo* in humans and in the atherosclerotic apolipoprotein E-deficient mice. Dietary supplementation of PJ rich in polyphenols to patients with severe carotid artery stenosis or to atherosclerotic mice resulted in a significant inhibition in the development of the atherosclerotic lesions, and this may be attributed to the protection of lipids in arterial wall as well as in serum LDL against oxidation. The preferred pomegranate product in terms of biological potency and consequent health benefits is the pomegranate juice (PJ) from the whole fruit. Since combination of antioxidants, as exist in PJ can provide a wider range of free radicals scavenging capacities than an individual antioxidant, clinical and nutritional studies in humans should be directed towards the use of combinations of several types of dietary antioxidants, including combinations of flavonoids together with other nutritional antioxidants, such as vitamin E and carotenoids. It is also important to use reliable biological markers of oxidative stress and to identify populations suitable for antioxidant treatment, as antioxidants treatment may be beneficial only in subjects which are under oxidative stress.

## Abbreviations

PJ: Pomegranate juice
LDL: Low density lipoprotein
HDL: High density lipoprotein
PON1: Paraoxonase 1
PON2: Paraoxonase 2
CHD: Coronary heart disease
CAS: Carotid artery stenosis
E^0^: Apolipoprotein E deficient
CIMT: Carotid intima media thickness
PSV: Peak systolic velocity
EDV: End diastolic velocity
TEAC: Trolox equivalent antioxidant capacity
ORAC: Oxygen radical absorbance capacity
DPPH: 2,2-Diphenyl-1-picrylhydrazyl
FRAP: Ferric reducing antioxidant power
AAPH: 2.2'-Azobis, 2-amidinopropane hydrochloride
TAS: Total antioxidant status
SH: Sulfhydryl
LPDS: Lipoprotein deficient serum
GAE: Gallic acid equivalents
GA: Gallic acid
PON1KO: PON1 Knockout
Ox-LDL: Oxidized LDL
MPM: Mouse peritoneal macrophages
NF*κ*-B: Nuclear factor kappa-B
HMGCoA-3: Hydroxy-3-methylglutaryl coenzyme A
HMDM: Human monocytes derived macrophages
DGAT1: Diacylglycerol acyltransferase 1
TPT: Total pomegranate tannins
PBP: Pomegranate liquid by-product
POMxp: Pomegranate fruit powder by-product
RNS: Reactive nitrogen species
ROS: Reactive oxygen species
TBARS: Thiobarbituric acid reactive substances
DCFH- 2′, 7′: Dichlorofluorescin diacetate.
